# Psychological distress and wellbeing as mediators between anti-mattering (feelings of insignificance) and irritability among Lebanese adolescents: A cross-sectional study

**DOI:** 10.1371/journal.pone.0334725

**Published:** 2025-10-14

**Authors:** Michelle Abi Karam, Rabih Hallit, Diana Malaeb, Fouad Sakr, Mariam Dabbous, Feten Fekih-Romdhane, Sahar Obeid, Souheil Hallit

**Affiliations:** 1 School of Medicine and Medical Sciences, Holy Spirit University of Kaslik, Jounieh, Lebanon; 2 Department of Infectious Disease, Bellevue Medical Center, Mansourieh, Lebanon; 3 Department of Infectious Disease, Notre Dame des Secours, University Hospital Center, Byblos, Lebanon; 4 College of Pharmacy, Gulf Medical University, Ajman, United Arab Emirates; 5 School of Pharmacy, Lebanese International University, Beirut, Lebanon; 6 Department of Psychiatry “Ibn Omrane”, The Tunisian Center of Early Intervention in Psychosis, Razi hospital, Manouba, Tunisia; 7 Faculty of Medicine of Tunis, Tunis El Manar University, Tunis, Tunisia; 8 School of Arts and Sciences, Social and Education Sciences Department, Lebanese American University, Jbeil, Lebanon; 9 Department of Psychology, College of Humanities, Effat University, Jeddah, Saudi Arabia; 10 Applied Science Research Center, Applied Science Private University, Amman, Jordan; University of Diyala College of Medicine, IRAQ

## Abstract

**Background:**

This study examined the mediating effect of psychological distress and wellbeing in the association between anti-mattering and irritability among a sample of Lebanese adolescents—a topic that remains largely underexplored in adolescent mental health research.

**Methods:**

A cross-sectional study was conducted during November 2023 and included 763 adolescents currently residing in Lebanon (mean age 16.08 ± 1.74 years, 37.6% males and 62.4% females) recruited from all Lebanese governorates using a snowball sampling technique. Data were collected via an online questionnaire and analyzed using the PROCESS Macro for mediation analysis.

**Results:**

The findings indicated that both psychological distress (β = .39, BootSE = .04, 95% CI [.33,.46]) and wellbeing (β = .07, BootSE = .02, 95% CI [.03,.12]) partially mediated the relationship between anti-mattering and irritability. Adolescents with higher levels of anti-mattering reported greater distress and reduced wellbeing, both of which were associated with increased irritability.

**Conclusion:**

Our results highlight the psychological relevance of anti-mattering in adolescence and suggest that addressing feelings of insignificance may play a key role in managing emotional dysregulation. Mental health practitioners and educators should consider developing culturally sensitive interventions that target feelings of anti-mattering, enhance wellbeing, and reduce psychological distress. School-based programs promoting emotional support and social inclusion may prove especially beneficial. Future studies should investigate these associations longitudinally and across varied cultural contexts to better inform prevention and intervention strategies.

## Introduction

Irritability is defined as a heightened sensitivity to provocation, and is marked by interindividual differences in anger susceptibility, low frustration tolerance, as well as a tendency toward excessive emotional or aggressive responses to minimal stimuli [1,2]. It can manifest in a phasic form which consists of behavioral outburst of intense anger and a tonic form which is characterized by a persistent grumpy or angry mood [3]. A previous study reported that at any given time during childhood/adolescence, 51.4% of participants reported phasic irritability, 28.3% reported tonic irritability, and 22.8% reported both [4]. Adolescence, defined as an age between 10–19 years [5], is a period of notable social, emotional, and physical changes that can both influence and be influenced by changes in irritability [6]. In addition, irritability is a symptom of many psychiatric conditions that arise or intensify during this phase, resulting in many lasting difficulties in adulthood [6,7]. Yet despite its clinical importance, little is known about the mechanisms through which social-emotional factors such as anti-mattering contribute to adolescent irritability. It is important to identify irritability as it is a prominent attribute of various types of psychological pathologies [8]. Furthermore, irritability may increase risk taking behaviors including substance abuse in adolescence [9]. Many risk factors such as child psychopathology, performance, temperament, parental psychopathology, and the psychosocial environment, have been shown to foster the development of irritability [8]. A previous study also demonstrated a link between anti-mattering and irritability, which could, as well, act as a risk factor [10].

Throughout history, humans have been driven by an inherent desire for social value, often referred to as the need for significance [11]. This need often pushes humans to seek recognition and validation from their social groups influencing behaviors like maintaining relationships that fulfills the desire for belonging and self-worth [12]. The desire becomes particularly pronounced during adolescence where, according to Erik Erikson, adolescents specifically require recognition from others as it is a crucial process for identity development [13]. Yet although adolescence is a time when recognition and relational affirmation are developmentally essential, little research has examined how the absence of such validation—what is referred to as anti-mattering—may compromise emotional security and wellbeing during this stage [14].

As humans, our need for significance is closely related to the concept of mattering, which can severely affect our wellbeing, both positively and negatively [15]. While believing that one matter can provide considerable protection, feeling that they do not can be destructive to their wellbeing [16].

Anti-mattering is characterized by a profound sense of insignificance, exclusion, invisibility, and being unseen by others [17]. This construct reflects an individual’s perception of lacking importance in social relationships [17]. Beyond a sense of neglect, anti-mattering threatens core psychological needs by eroding adolescents’ perceptions of social relevance and belonging, placing them at increased risk for emotional distress, anxiety, depression, and irritability [10]. A previous study revealed a connection between feelings of not mattering and a higher level of irritability; hence, the frustration of unmet needs for significance contributes to increased emotional reactivity [10]. The sense of not mattering has also been associated with increased irritability because of the negative self-concepts from repeated negative interpersonal encounter leading to sustained depressive cycles [18]. Rosenberg (1985) also reported that adolescents who felt they did not matter to their parents showed greater emotional reactivity and irritability, suggesting early links between low perceived significance and affective instability [19]. Likewise, anti-mattering was linked to increased feelings of loneliness, reduced self-esteem, and a lack of social support, which are all linked to irritability [20,21]. Flett (2025) further emphasized that chronic feelings of anti-mattering may contribute to youth aggression, withdrawal, and emotional dysregulation—highlighting the need to examine this construct in relation to irritability [22]. By exacerbating emotional dysregulation, anti-mattering could promote the development of irritability [21]. Other possible mechanisms linking anti-mattering to irritability, which the current study focuses on, are psychological distress and wellbeing.

Psychological distress and wellbeing were hypothesized to be plausible mediators based on literature suggesting that these factors could be affected by anti-mattering, and could lead to irritability. Feelings of anti-mattering have been linked to loneliness and distress due to the intense feelings of social disconnection [20]. Psychological distress is defined as a unique emotional discomfort resulting from the inability to effectively manage a stressor, often manifesting as symptoms of depression and anxiety following exposure to a stressful event [23]. Previous studies have shown that anti-mattering is implicated in extensive distress and dysfunction emphasizing how feeling unvalued, invisible, and ignored can contribute to emotional upset [20]. Moreover, the fact that anti-mattering was linked to social anxiety and loneliness could explain the increase in feelings of distress [18,21]. Additionally, the stress that results from anti-mattering can lead to difficulties in the management of emotions and poor emotional regulation skills which in turn increases the likelihood of emotional outbursts [21]. On the other hand, it has been shown that psychological distress could lead to irritability by fostering stressful environments that lead to an overwhelming of emotions [24]. Distress can also lead to increased emotional reactivity and frustration intolerance, which can cause individuals to experience irritability [25].

The construct of wellbeing has been defined as an equilibrium between psychological, social, and physical resources and challenges; however, it can be impaired when the former outweighs the latter. As reported by the Centers for Disease Control and Prevention (CDC), wellbeing comprises positive emotions, fulfillment in life, and efficient functioning [26]. Previous studies revealed a strong negative link between anti-mattering and wellbeing with evidence suggesting that feeling like you do not matter can be detrimental to one’s wellbeing [16]. This correlation between anti-mattering and lower levels of wellbeing could be explained by the different attachment style adopted by people who feel they like they do not matter [16,20] Overall, the aforementioned associations suggest that psychological distress and wellbeing may act as a mediator between anti-mattering and irritability.

Unlike most previous studies, which have focused on general distress or depressive symptoms, this study investigates how both distress and wellbeing mediate the impact of anti-mattering on irritability [27]. Exploring anti-mattering feelings in Lebanese adolescents is particularly important due to the country’s unique cultural and socio-economic context. Lebanese society is deeply rooted in collectivism, where family and social bonds are highly valued [28,29]. While this cultural emphasis on connectedness can serve as a protective factor, it may also amplify feelings of exclusion when adolescents perceive themselves as insignificant or disconnected [27,30], Adolescence is a critical developmental stage characterized by identity formation and a growing need for social belonging. The transition from childhood to adulthood heightens vulnerability to anti-mattering, particularly in a collectivist society where social validation is emphasized. The inability to establish a sense of significance within one’s family and peer groups may contribute to increased irritability and emotional distress. Furthermore, Lebanon’s ongoing socio-economic challenges add an additional layer of complexity to adolescent well-being [31]. For that the Household Crowding Index (HCI) was included as a demographic variable to account for socioeconomic differences, as it is linked to emotional dysregulation and stress. In Lebanon, household crowding remains a significant public health concern, disproportionately affecting adolescents in lower-income communities where personal space is scarce, and psychosocial stressors accumulate [32]. Greater levels of crowding have been linked to increased psychological distress, irritability, and sleep disturbances in adolescents, particularly those from low-income households [33,34]. Beyond economic difficulties, factors such as political crises, widespread instability, and security concerns have been identified in a previous study as major contributors to increased psychological distress in Lebanon [35]. This research is among the first to assess anti-mattering in relation to irritability in adolescents, and uniquely does so within an Arab cultural context where social belonging and interpersonal validation are central to identity formation.

Therefore, this study aims to assess the mediating effect of psychological distress and wellbeing in the association between anti-mattering and irritability within a sample of Lebanese adolescents. We hypothesize that adolescents who perceive greater levels of anti-mattering will report higher psychological distress and lower wellbeing. Hence, these factors are anticipated to mediate the association between anti-mattering and increased irritability, reflecting the indirect pathways through which feelings of insignificance may affect mood states.

## Methods

### Ethics approval and consent to participate

The Ethics and Research Committee of the School of Pharmacy at the Lebanese International University approved this study protocol (2023RC-033-LIUSOP). Informed consent was obtained from the parent and/or legal guardian for participants under age 16 before filling the survey; submitting the form online was considered equivalent to obtaining a written informed consent.

### Study design

This was a cross-sectional study performed in November 2023, intentionally timed to minimize the confounding effect of academic stress from examinations [36]. Participants were eligible if they were Lebanese, aged 14–18 years, residing in Lebanon, currently enrolled in school and from all Lebanese governorates. To screen participants for eligibility, we included a self-reported age question at the start of the online survey. Individuals who did not wish to participate or those who did not fit the adolescent age category were excluded. In order to enhance the statistical power and enable comprehensive subgroup analyses, a large sample size was selected.

Inclusion criteria were: (1) currently residing in Lebanon, (2) aged 15–19 years, (3) access to the internet and a device to complete the online survey, and (4) proficiency in reading Arabic. Participants were recruited from both public and private schools through online outreach.

The questionnaire was administered anonymously to ensure participant privacy and encourage honest response. Recruitment was conducted primarily through WhatsApp, Instagram and Facebook which are the most widely used communication platform among Lebanese adolescents. Snowball sampling was employed to reach more individuals, as research resources are limited in Lebanon and financial costs of interviewer-administered methods cannot be afforded.

A soft copy of the questionnaire was created via google forms. To ensure ethical compliance, participants were briefed on the study’s objectives with all the necessary information, including the option to withdraw at any point. Informed consent was obtained before participation. The study protocol was approved by The Ethics and Research Committee of the School of Pharmacy at the Lebanese International University, ensuring adherence to ethical guidelines. After that, participants were instructed to recruit acquaintances of the same age range. Participation was voluntary and uncredited.

### Minimal sample size calculation

A minimal sample of 125 participants was deemed essentially using the formula suggested by Fritz and MacKinnon [37]: “$n=\frac{L}{\hat{f}\text{2}}+k+\text{1}$” where L = 7.85 for an α error of 5% and power β = 80%, f = 0.26 for an eﬀect size of small to medium, and k = 8 variables to be included in the model.

### The Questionnaire

The Arabic questionnaire assessed the sociodemographic characteristics of the involved participants (age, gender and Household Crowding Index (HCI)). The HCI is determined by dividing the total household population (excluding new-borns) by the number of rooms in the residence, except the kitchen [32], alongside the following scales:

***The Anti-Mattering Scale***, validated in Arabic [38], is composed of 5 items measuring an individual’s perceived insignificance to others, such as “How much do you feel like you don’t matter?”, “How often have you been treated in a way that makes you feel like you are insignificant?”, “To what extent have you been made to feel like you are invisible?”, “How much do you feel like you will never matter to certain people?”, and “How often have you been made to feel by someone that they don’t care what you think or what you have to say?” [16,20]. The items are evaluated on a scale extending from 1 (not at all) to 4 (a lot) with higher scores denoting a higher level of anti-mattering (current Cronbach’s α = .87).

***The Brief Irritability Test (BITe)*** [39], validated in Arabic [40]. consists of five-item scale where each item is rated on a 6-point Likert scale ranging from 1 (never) to 6 (always). Responses to all items are summed to yield a total score, with higher scores corresponding to greater levels irritability (current Cronbach’s α = .87).

***The World Health Organization – Five wellbeing Index*** (***WHO-5)***, validated in Arabic [41], is a 5-item scale that evaluates subjective psychological wellbeing (“I have felt cheerful in good spirits, I have felt calm and relaxed, I have felt active and vigorous, I woke up feeling fresh and rested, My daily life has been filled with things that interest me”). Each item is scored on a 5-point Likert scale. The total score, ranging from 0 to 25, is multiplied by 4 to get the final score, with 0 reflecting the worst possible wellbeing and 100 meaning the best possible wellbeing [42]. (current Cronbach’s α = .92)

***The Depression Anxiety Scale-8 (DASS-8),*** validated in Arabic among adolescents [43] and adults [44] is a compacted version of DASS-21 and involves 8 items of 3 subscales: depression (3 items), anxiety (3 items) and stress (2 items) [44]. The items are rated on a 4-point scale, extending from 0 (did not apply to me at all) to 3 (applied to me very much or most of the time). The overall score of the DASS-8 ranges from 0 to 24, while the subscale scores range from 0 to 9, 0–9, and 0–6, respectively. Greater scores denote a heightened level of symptoms (current Cronbach’s α = .88).

No additional construct validity testing or pilot study was performed in this sample. However, all scales have been validated in prior research, including in Arabic-speaking populations.

### Statistical analysis

Statistical analysis was performed via the SPSS software v.25. The irritability score was normally distributed as its skewness and kurtosis vary between ±2 [45]. In order to compare two means, Student t test was used along with the Pearson test used to correlate two continuous variables. Mediation analysis was performed using PROCESS MACRO v.3.4 for SPSS model 4 [46]; four pathways drawn from this analysis: pathway A from anti-mattering to psychological distress/wellbeing, pathway B from psychological distress/wellbeing irritability, Pathways C and C’ indicating the total and direct effects of anti-mattering on irritability respectively. The bootstrapping method with 5,000 resamples was used to generate bias-corrected 95% confidence intervals (CIs). Mediation analysis was considered significant if the CI did not include zero. We adjusted the mediation models for covariates identified in the bivariate analyses (*p* < 0.25), including age, gender and household crowding index. Nagelkerke R^2^ values were calculated to estimate the effect size for each model, with values between 0.02–0.13 reflect small effect, whereas values between 0.13–0.26 and ≥ 0.26 indicate medium and large effect respectively [47]. Before conducting the mediation analysis, linearity between variables was confirmed via scatterplots. Normality of residuals was evaluated using Q-Q plots; no significant deviations were observed. Homoscedasticity was checked by inspecting plots of standardized residuals versus predicted values. Multicollinearity was assessed through the Variance Inflation Factor values, which were < 10, indicating no multicollinearity. *P* < 0.05 was recognized as statistically significant.

## Results

### Participant characteristics

A total of 793 adolescents (mean age 16.08 ± 1.74 years) participated, with 62.4% females (n = 495) and 37.6% males (n = 298). Other characteristics can be found in Table 1.

**Table 1:** Description of the sample (n = 793).

Gender	
Males	298 (37.6%)
Females	495 (62.4%)
Age (in years)	16.08 ± 1.74
Household crowding index (person/room)	1.23 ± 2.14
Irritability	13.39 ± 4.78
Wellbeing	20.54 ± 5.38
Anti-mattering	10.99 ± 3.60
Psychological distress	9.93 ± 5.17

### Bivariate analysis of factors associated with irritability

A higher mean irritability score was found in females compared to males (13.78 ± 4.95 vs 12.76 ± 4.41, t(791) = −2.91; p = .004, Cohen’s d = 0.213). Moreover, a higher irritability score was significantly associated with higher anti-mattering and psychological distress scores, and lower wellbeing scores (Table 2).

**Table 2:** Pearson correlation matrix.

	Irritability	Anti-mattering	Wellbeing	Psychological distress	Age	Household crowding index
1. Irritability	1					
2. Anti-mattering	.47***	1				
3. Wellbeing	−.65***	−.37***	1			
4. Psychological distress	.66***	.53***	−.50***	1		
5. Age	.03	−.02	−.06	.001	1	
6. Household crowding index	−.004	−.06	.04	−.05	−.02	1

***p < .001

### Mediation analysis

**Fig 1:**
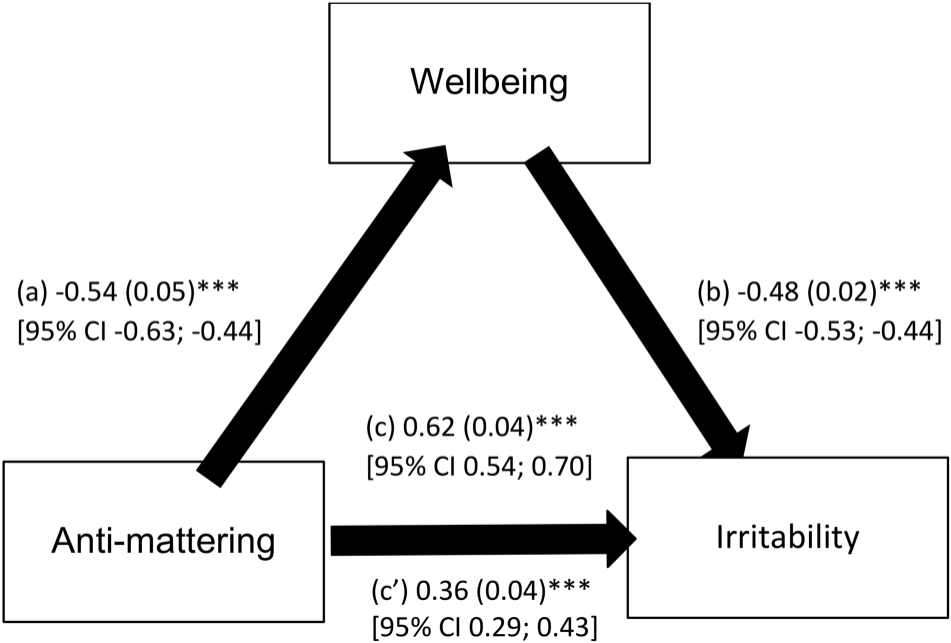
Mediation analysis of wellbeing between anti-mattering and irritability. (a) Relation between anti-mattering and wellbeing (the mediator) (R^2^ = 0.145); (b) Relation between wellbeing and irritability (R^2^ = 0.483); (c) Total effect of anti-mattering on irritability (R^2^= 0.229); (c’) Direct effect of anti-mattering on irritability. Numbers are presented as regression coefficients (standard error). ***p < .001.

In the mediation analysis, adjustments were made over gender. Wellbeing partially mediated the association between anti-mattering and irritability since the indirect effect of wellbeing between anti-mattering and irritability was significant (Beta = .26, BootSE = .03, 95% CI.20,.33). Higher anti-mattering was significantly correlated with lower wellbeing (Standardized Beta = −0.36; large effect size: R^2^ = .274), and higher wellbeing was considerably associated with lower irritability (Standardized Beta = −0.55; large effect size: R^2^ = .577). Finally, higher anti-mattering was significantly associated with higher irritability (Standardized Beta for the total effect = 0.47; large effect size: R^2^ = .457) (Fig 1). These results confirm the partial mediation of wellbeing between anti-mattering and irritability, with an overall model R^2^ of.577, signaling that 57.7% of the variance in irritability is accounted for by wellbeing.

**Fig 2:**
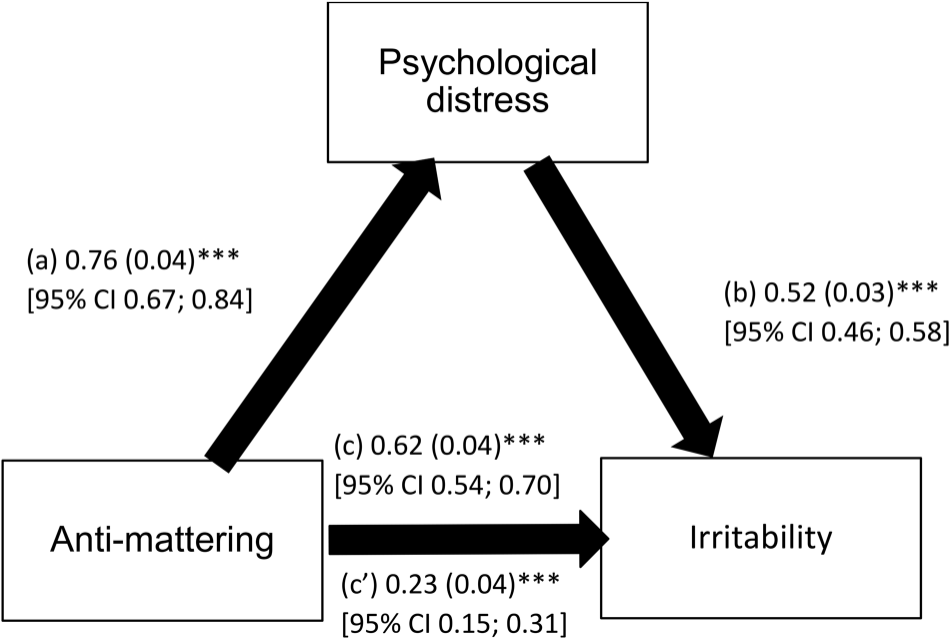
Mediation analysis of psychological distress between anti-mattering and irritability. (a) Relation between anti-mattering and psychological distress (the mediator) (R^2^ = .282); (b) Relation between psychological distress and irritability (R^2^= .457); (c) Total effect of anti-mattering on irritability (R^2^ = .229); (c’) Direct effect of anti-mattering on irritability. Numbers are displayed as regression coefficients (standard error). ***p < .001.

Psychological distress partially mediated the association between anti-mattering and irritability since the indirect effect of psychological distress between anti-mattering and irritability was significant (Beta = .39, BootSE = .04, 95% CI.33,.46). Higher anti-mattering was significantly associated with increased psychological distress (Standardized Beta = 0.53; large effect size: R^2^ = .282), and higher psychological distress was significantly linked to higher irritability (Standardized Beta = 0.56; large effect size: R^2^ = .457). Finally, higher anti-mattering was significantly correlated with higher irritability (Standardized Beta for the total effect = 0.47; moderate effect size: R^2^ = .229) (Fig 2). These results prove the partial mediation of psychological distress between anti-mattering and irritability, with an overall model R^2^ of.457, indicating that 45.7% of the variance in irritability is accounted for by psychological distress.

## Discussion

The objectives of this research were to explore the relationship between anti-mattering and irritability, along with the mediation effect of wellbeing and distress in this relationship among Lebanese adolescents. Our results revealed a substantial significant positive direct effect of anti-mattering on irritability. Furthermore, the results of the mediation analysis revealed that wellbeing and distress had an important indirect effect on the association between anti-mattering and irritability. The household crowding index was included as a demographic variable to provide contextual information on participants’ living conditions. Furthermore, the correlation between irritability and age was statistically non-significant which shows that irritability represents a stable trait in adolescence, independent of chronological age.

### The association between anti-mattering and irritability

As for the direct effect, and as expected, the findings indicated that anti-mattering was associated with irritability. This resonates with previous literature suggesting that adolescents who feel insignificant often experienced low self-esteem and low self-worth, which can lead to increased irritability as they become more vulnerable and sensitive to criticism [14]. Feelings of anti-mattering can lead to significant stress, which in turn can cause dysregulation of emotion resulting in irritability [48]. Feeling unimportant can also increase one’s sensitivity to rejection leading to irritability and overreaction to perceived threats from neglect [49]. Anti-mattering feelings can also be the reason individuals lose purpose in life, which can cause existential frustration and irritability [49]. Anti-mattering was also proven to be linked to neuroticism and emotional instability possibly due to the low levels of self-liking and self-esteem [20]. Anti-mattering has also been associated with insecure attachment styles—such as anxious or avoidant patterns—which may contribute to emotional dysregulation and, in turn, to increased irritability [20].

### Psychological distress as a mediator

Consistent with earlier findings, anti-mattering was associated with higher psychological distress, as feeling unvalued and invisible can lead to emotional upset [16,20]. Such perceptions may weaken a sense of belonging and self-worth, increasing vulnerability to distress [20]. While our cross-sectional design does not allow for conclusions about how these effects develop over time, the observed association supports the role of psychological distress as a potential pathway linking anti-mattering to irritability [50]. The relationship between anti-mattering and irritability appears to be partially mediated by psychological distress, as indicated by our mediation analysis. This suggests that individuals who feel insignificant experience heightened psychological distress, which in turn amplifies irritability. From a short-term perspective, anti-mattering may trigger immediate emotional distress due to acute social exclusion or perceived rejection, leading to heightened irritability [15]. Increased levels of psychological distress also significantly correlated with higher irritability. While this association was shown to be significant here, other studies found psychological distress to be a nonspecific syndrome from which irritability is part of [51,52]. Psychological distress appeared both as an internal emotional state and as a factor disrupting interpersonal functioning, amplifying irritability [51].

It is worth noting that some discrepancies may exist in the literature concerning the role of psychological distress in adolescent irritability. While our findings indicate that psychological distress partially mediates the association between anti-mattering and irritability, other studies have conceptualized distress as a broad internalizing syndrome—encompassing irritability itself as a symptom—rather than a causal pathway. For example, distress is often included as part of depressive or anxiety disorders in major classification systems such as the DSM-5, where irritability is considered a core component rather than an outcome. Similarly, a previous study has shown that irritability may co-occur with other emotional symptoms without a clearly defined directional pathway [53]. These conceptual differences may explain why some studies did not observe a mediation effect. Our study, however, adopts a socio-emotional perspective wherein perceived insignificance contributes to internal distress, which in turn manifests as external irritability. This interpretation aligns with existing models describing the links between social experiences and mood states in adolescence and supports the need for future longitudinal designs to confirm causal sequences [54].

Altogether, our findings and observations suggest that psychological distress could act as an intermediary factor between anti-mattering and irritability. The feeling that others do not matter can lead to emotional distress which in turn can worsen feelings of irritability due to the psychological turmoil and negative appraisal linked with distress [55,56]. This mediating impact of psychological distress on the association between anti-mattering and irritability emphasizes the importance of targeting this important variable when aiming to address irritability in adolescents.

### Wellbeing as a mediator

Our results revealed that wellbeing was acted as a mediator between anti-mattering and irritability. This signifies that wellbeing might play the role of an intermediate factor that underscores the connection between anti-mattering and irritability. This denotes that individuals with intense feelings of anti-mattering may be more inclined to have disrupted overall wellbeing, which in turn, could increase the risk of developing irritability by dysregulating emotions [20,57]. Consistent with our findings, anti-mattering was shown to display a significant negative relationship with wellbeing [20]. Wellbeing has been linked to a positive mood and robust coping skills contributing to greater resilience and lessening the probability of manifesting irritability in response to stressors [58]. Aligned with our results, numerous recent studies identified a strong connection between distress and irritability [59,60]. Even though this association was established in previous researches in reverse direction to this study (the effect of irritability on wellbeing was the assessed association) [59,60]. It has been shown that greater levels of irritability were linked to lesser wellbeing and life satisfaction as well as higher perceptions of negative social relationships [60]. Another study found that wellbeing does not only affect individuals directly but also indirectly through coping styles [59]. Consequently, having a positive coping style enhances subjective wellbeing experiences, while having a negative coping style makes setbacks more intense and reduces wellbeing [59]. Individuals who engage in adaptive coping strategies, such as problem-solving and emotional acceptance, tend to maintain higher well-being and exhibit greater emotional stability. Conversely, maladaptive coping strategies, such as avoidance and rumination, can exacerbate distress and heighten irritability levels [61]. One study even suspected a bidirectional relationship between wellbeing and irritability [62]. Individuals with high psychological wellbeing tend to have better emotional regulation skills leading to emotional stability and a better overall management of negative emotions thus decreasing the likelihood of irritability [63].

In sum, our results might have important implications for designing help strategies aiming to increase wellbeing, which will then act as a protective factor [64]. However, it is crucial to acknowledge that discrepancies in individual’s experiences and outcomes can exist, and that might be significantly due to genetic, environmental, cultural and personal factors predisposing individuals to certain feelings and emotions.

### Theoretical implications

The findings of this study contribute to existing theory by underlining how anti-mattering affects adolescent irritability through key emotional processes. This supports Erikson’s theory that adolescence is marked by a need for social validation and extends Rosenberg’s and Flett’s work by showing that the absence of perceived significance (anti-mattering) increases vulnerability to psychological distress and disrupts wellbeing. By identifying distress and wellbeing as mediators, this study deepens our understanding of how anti-mattering leads to internalizing symptoms during adolescence.

### Strengths and limitations

The study’s reliability is reinforced by many key strengths that magnify its contributions. The inclusion of a large sample of 763 participants and the use of well-validated scales to evaluate study variables increase the statistical power and reliability of our results. Nevertheless, our research is not without limitations. First, the cross-sectional design restricts our capacity to infer causal and temporal relationships among the variables studied and the high percentage of females may affect the generalizability. Second, it was based on self-reported questionnaires, which may lead to social desirability bias. Moreover, the use of the snowball sampling technique via social media to recruit participants may not adequately embody the diversity of the wider population. A potential information bias may be present since participants may not provide sincere and true responses to the questions. Additionally, the skewed distribution of the household crowding index reflects different living conditions among participants. And, while this study focused on psychological variables, it did not explicitly account for cultural influences such as collectivism. Finally, the overrepresentation of female participants (62.4%) limits the capability to generalize findings to male adolescents. Research shows that females have a tendency to report greater levels of psychological distress and social sensitivity, which may partially explain their heightened irritability and distress scores [65]. Additionally, in collectivist societies like Lebanon, social belonging and familial validation are central to adolescent wellbeing, potentially amplifying the psychological consequences of anti-mattering [12] And while the Household Crowding Index was included as a proxy for socioeconomic status, other variables such as parental education and perceived social support were not assessed, representing a limitation.

### Clinical implications

Understanding how anti-mattering feelings contribute to irritability in adolescents holds important clinical relevance, particularly in culturally specific contexts like Lebanon. First, the findings suggest that clinicians working with adolescents should screen for anti-mattering feelings, especially in cases presenting with heightened irritability. Anti-mattering, a feeling of insignificance or being invisible, may not be routinely assessed in mental health evaluations, yet it appears to play a fundamental role in emotional dysregulation.

Second, the mediation results indicate that psychological distress and reduced wellbeing partially justify the link between anti-mattering and irritability. This highlights the need for intervention strategies that directly address these mediating factors. Clinicians may consider using evidence-based modalities such as cognitive behavioral therapy (CBT), acceptance and commitment therapy (ACT), or mindfulness-based interventions that specifically target distress tolerance and emotion regulation. Such interventions may not only reduce distress but also enhance a sense of mattering and self-worth, which could reduce irritability.

Third, these results emphasize the significance of preventative, school-based, and community-level programs that promote social inclusion and belonging among adolescents. Programs focused on improving peer relationships, fostering teacher-student connectedness, and reducing bullying may serve as upstream strategies to buffer the emergence of anti-mattering and its psychological consequences.

Finally, the research highlights the importance of early identification and intervention in culturally sensitive ways. Given the social and political stressors faced by Lebanese youth, mental health practitioners should be attuned to the sociocultural context that may amplify feelings of insignificance. Integrating these insights into clinical practice may improve treatment outcomes and adolescent wellbeing. Given the cross-sectional design, results should be inferred as exploratory and associative, not causal. However, the results suggest that school-based mental health programs and peer support initiatives tailored to Lebanese adolescents could play an important role in reducing emotional distress and fostering wellbeing.

## Conclusion

This study reveals that anti-mattering is significantly linked to adolescent irritability, with psychological distress and wellbeing acting as partial mediators. Adolescents who perceive themselves as insignificant are more prone to emotional distress and reduced wellbeing, both of which contribute to increased irritability. These findings highlight anti-mattering as a potential early psychological risk factor in youth, suggesting the value of targeted interventions—such as cognitive behavioral therapy and mindfulness-based programs—to enhance self-worth and emotional regulation. Although the cross-sectional design prohibits causal conclusions, the strong associations observed provide a foundation for subsequent studies. Longitudinal and cross-cultural studies are indispensable to delve into temporal relationships, assess cultural variability, and identify potential moderators such as gender, social support, or peer dynamics. Incorporating anti-mattering into adolescent mental health screening could help guide early intervention strategies and improve clinical outcomes across diverse populations.
